# Underwater Wireless Sensor Networks with RSSI-Based Advanced Efficiency-Driven Localization and Unprecedented Accuracy

**DOI:** 10.3390/s23156973

**Published:** 2023-08-05

**Authors:** Kaveripakam Sathish, Ravikumar Chinthaginjala, Wooseong Kim, Anbazhagan Rajesh, Juan M. Corchado, Mohamed Abbas

**Affiliations:** 1 School of Electronics Engineering, Vellore Institute of Technology, Vellore 632014, India; sathishkvit58@gmail.com (K.S.); cvrkvit@gmail.com (R.C.); 2 Department of Computer Engineering, Gachon University, Seongnam 13120, Republic of Korea; wooseong@gachon.ac.kr; 3 School of Electrical and Electronics Engineering, SASTRA University, Thanjavur 613401, India; rajeshtechece@gmail.com; 4 BISITE Research Group, University of Salamanca, 37007 Salamanca, Spain; corchado@usal.es; 5 Air Institute, IoT Digital Innovation Hub, 37188 Salamanca, Spain; 6 Department of Electronics, Information and Communication, Faculty of Engineering, Osaka Institute of Technology, Osaka 535-8585, Japan; 7 Electrical Engineering Department, College of Engineering, King Khalid University, Abha 61421, Saudi Arabia

**Keywords:** localization, UWSN, mean estimation error, RSSI, TOA, TDOA

## Abstract

Deep-sea object localization by underwater acoustic sensor networks is a current research topic in the field of underwater communication and navigation. To find a deep-sea object using underwater wireless sensor networks (UWSNs), the sensors must first detect the signals sent by the object. The sensor readings are then used to approximate the object’s position. A lot of parameters influence localization accuracy, including the number and location of sensors, the quality of received signals, and the algorithm used for localization. To determine position, the angle of arrival (AOA), time difference of arrival (TDoA), and received signal strength indicator (RSSI) are used. The UWSN requires precise and efficient localization algorithms because of the changing underwater environment. Time and position are required for sensor data, especially if the sensor is aware of its surroundings. This study describes a critical localization strategy for accomplishing this goal. Using beacon nodes, arrival distance validates sensor localization. We account for the fact that sensor nodes are not in perfect temporal sync and that sound speed changes based on the medium (water, air, etc.) in this section. Our simulations show that our system can achieve high localization accuracy by accounting for temporal synchronisation, measuring mean localization errors, and forecasting their variation. The suggested system localization has a lower mean estimation error (MEE) while using RSSI. This suggests that measurements based on RSSI provide more precision and accuracy during localization.

## 1. Introduction

Underwater wireless sensor networks (UWSNs) are networks comprised of sensors positioned in or near water that are capable of communicating with one another and sharing data. Environmental monitoring, underwater surveillance, and ocean exploration are just a few of the possibilities for these networks [1]. The problems associated with identifying sensor nodes in an underwater environment are a significant hurdle for UWSNs. To overcome the difficulty of localization and achieve a level of precision previously unattainable in UWSNs, a technology known as RSSI-based advanced efficiency-driven localization was created [2,3,4]. RSSI is a statistic that evaluates the strength of a received radio signal and can be used to calculate the distance between two nodes. The placements of the sensor nodes are determined using the RSSI values of the signals received by the nodes surrounding them in this manner [5].

The RSSI-based advanced efficiency-driven localization approach optimises the localization process by utilising time-saving strategies and protocols. This results in improved localization accuracy. In order to accomplish precise and cost-effective localization, these algorithms take into account a number of aspects, including power consumption, communication overhead, and network scalability. Using cutting-edge algorithms makes the localization process more durable and dependable in difficult situations, such as underwater environments with low bandwidth, significant propagation loss, and the influence of multipath effects [6,7,8].

This localization technology’s exceptional accuracy is critical for a wide range of applications. Accurate localization, for example, enables high-resolution monitoring of subsea habitats and the migration of marine organisms in the context of environmental monitoring [9]. The precise positioning of sensor nodes used in underwater surveillance allows for the early detection of any anomalies or invasions in underwater infrastructure such as pipelines or offshore locations. This increases the likelihood of early detection of any potential dangers. This technique of localization also contributes to the advancement of marine research. Researchers can uncover new ecosystems, underwater archaeological sites, or geological formations more easily if they construct exact maps of underwater regions and correctly place sensor nodes [10,11,12,13].

### 1.1. Problem Statement

The environment of a body of water poses a unique set of obstacles for wireless communication and localization. Water’s high attenuation, multipath propagation, and the confined bandwidth of aquatic environments all conspire against the efficiency and precision of wireless sensor networks [14,15,16]. Finding the precise locations of sensor nodes during the localization process is a difficult challenge made more difficult by the lack of dependable Global Positioning System (GPS) signals and the necessity for steady and trustworthy underwater positioning algorithms. So, in this study, we are proposing RSSI-based advanced efficiency-driven localization.

### 1.2. Motivation

Because of the specific obstacles that arise in underwater environments, wireless sensor networks have had to develop their localization strategies in order to circumvent underwater communication restrictions. The demand for more precise environmental monitoring, better underwater surveillance, and enhanced exploration capabilities drove the development of UWSNs with RSSI-based advanced efficiency-driven localization and unprecedented precision [17,18,19]. Another motivation is to improve exploring abilities. Accurate localization enables researchers to collect high-resolution data on marine life, ecosystems, and environmental conditions, which aids in the protection of these areas and the mitigation of natural disasters. Furthermore, UWSNs with precise positioning can efficiently monitor underwater structures and detect anomalies in real time. This contributes to the stability of critical infrastructure [20]. Finally, advances in localization technologies have substantially improved the accuracy of underwater mapping and navigation. As a result, previously unknown information about Earth’s geology, archaeology, and history have been revealed. These technological developments make UWSNs live longer and be more reliable by optimising their energy utilisation and ability to communicate with one another. This enables UWSNs to continue data collection and processing even in difficult underwater settings [21].

Because of the fluidity of water, the algorithms employed by UWSNs to estimate positions under the water may encounter significant challenges. Furthermore, the constituent nodes of the UWSN may have difficulty communicating with one another. Estimating the strength of a received signal with existing technology and hardware may appear to be a simple and low-cost process [22]. Underwater sensor nodes, on the other hand, have limited energy reserves, are difficult to repair, and are susceptible to water damage. The purpose of this study is to address the aforementioned issue front-on by presenting a mechanism for 3D localization of UWSNs using current RSSI data. Using data that has been edited and weighted is one technique for lowering the likelihood of making mistakes [23]. The method of least squares can be used to estimate the location of an unknown node in three-dimensional space if the indicated strategy is followed. The easiest way to accomplish this is to create a 3D model of the object’s position in the water. The proposed method enhances dependability, is less likely to be disrupted by water movement, and allows for more precise network node localization.

This manuscript makes the following contributions, which are most important:

➢ Investigation of Scaling Effect: The manuscript explores how object localization changes as the number of UWSNs grows. This analysis helps in understanding the impact of network size on the accuracy and effectiveness of object localization in UWSNs.
➢ Evaluation of Distance-based Localization Algorithms: The manuscript examines distance-based localization algorithms in the context of UWSNs. By evaluating these algorithms, the study provides insights into their suitability, performance, and limitations for underwater localization scenarios.
➢ Proposal of an Effective Localization Strategy: The manuscript proposes and recommends an acceptable localization strategy based on the desired RSSI data. This strategy aims to optimize object localization in UWSNs, taking into account the specific requirements and characteristics of the underwater environment.

The manuscript is structured into the following: Section 2 discusses the related works. Section 3 discusses the proposed network design and simulation settings of the underwater localization algorithms. Section 4 presents an examination of the simulation results. Section 5 contains an overview of the plan, followed by a series of final observations and future scope.

## 2. Related Works

Li et al. [24] described a TDOA-based localization algorithm majorization-minimization (T-MM) localization strategy. T-MM employs the majorization-minimization (MM) algorithm in concert with the TDOA technique for acoustic localization. Since subsequent iterations of the MM method depend on the beginning points, the T-MM system employs a gradient-based initial point technique. The squared position error bound (SPEB) expression is calculated utilising the equivalent Fisher information matrix (EFIM) and is used to evaluate the performance of the proposed T-MM localization method. Even in the presence of substantial underwater noise, simulation results demonstrate that the proposed T-MM method outperforms current localization algorithms in terms of accuracy and computing cost.

Arbula et al. [25] estimated AoA with great accuracy by utilising low-range infrared (IR) data in line of sight (LOS). The sensor provides a suitable option for localization. To avoid propagation issues, this solution uses a wireless sensor network (WSN). We put the method to the test in the tough context of grocery store cart movement. We built a proof-of-concept navigation system using an IR-AoA sensor prototype and WSN for cart localization, and a server-side and client application suite of smartphone and wristwatch apps. Four evaluation methods—empirical and simulated—were used to assess the localization efficiency of the proposed strategy. The study revealed that a centimetre of accuracy in a static one-dimensional setting corresponded to a metre of localization inaccuracy for a moving cart travelling at 140 cm/s in a two-dimensional setup. These findings demonstrate that supermarket localization precision and real-time navigation support may be achieved with widely available infrared technology and low-cost hardware components.

Ullah et al. [26] proposed methods for accurate underwater localization that are also effective in terms of their use of energy. These approaches are based on measurements of both distance and angle. The pinpointing of underwater nodes is a primary focus of the systems that have been developed, particularly MEEs. A considerable amount of modelling is required in order to compare the proposed systems to those of other organisations. The proposed designs are an improvement on the MEEs in terms of both their location and their energy use.

Sahota et al. [27] examined the difficulty of locating wireless sensor network nodes in above- and below-ground anchor nodes. Distance is measured by the time a signal travels from a sensor node to a satellite node or vice versa. Estimating a joint distribution of arrival times defines localization. The probability distribution of a signal’s arrival time is derived from the sensor nodes’ geographical locations using sophisticated statistical analysis. This lets signal arrival time express distribution. The network’s joint distribution of time-of-arrival variables is based on maximum likelihood estimations of node geographic coordinates. Sensitivity analysis evaluated the model and approach. This study investigates the disparity between soil complex permittivity and magnetic permeability estimates and measurements.

Dubrovinskaya et al. [28] investigated exploiting wideband arrays of opportunity to construct practical algorithms that can accurately detect and estimate direction of arrival, and estimate position. DoA and rough multilateration estimations remove spatial uncertainty caused by the array’s architecture. We simulate underwater noise and acoustic congestion to prove our strategy works. These simulations indicate that our method correctly calculates DoA and position and that chance arrays can beat well-constructed arrays.

Hayder et al. [29] used the three main methods for changeable transmission power-based sparsity-conscious energy-efficient clustering (CTP-SEEC) in UWSNs used before. These include the adaptive power control mechanism that converts to a suitable transmission power level (TPL) and deploys cooperation mobile sinks or autonomous underwater vehicles (AUVs) to acquire local information for WSN energy and data management efficiency (security) for WSN security. After comprehensive simulations and testing, the suggested approach is compared to other cutting-edge UWSN protocols, to validate it. The continuous environmental condition simulation showed that the recommended protocol performs well in network lifetime, packet delivery, and throughput.

Qin et al. [30] proposed the RSSI-based three-dimensional UWSN positioning algorithm as one solution. A stationary node weights the signal strength it receives from a mobile anchor node to compensate for water effects, improving range measurement. Based on the distance between the unlocated node and the anchor node, a location estimation model is merged with a three-dimensional underwater model using least squares to calculate the missing node’s location. The novel method improves three-dimensional underwater positioning algorithms and lowers the aquatic environment’s effect on range algorithm accuracy. According to simulations, the proposed strategy may reduce the undersea environment’s detrimental effects on positioning algorithms.

These studies contribute to the field of underwater localization by proposing novel techniques, improving accuracy and energy efficiency, addressing propagation challenges, and exploring the use of different technologies and algorithms for accurate positioning in UWSNs.

## 3. Proposed Network Design and Simulation Settings

The proposed algorithm for calculating the intensity of an incoming received-signal-strength-based ranging method aims to interpret the signal intensity from an anchor node at a specific distance using an unnamed node. In this scenario, the anchor node is movable and responsible for transmitting the signal. However, due to environmental factors, there is some data loss during the transmission process. As the distance increases, the unlocated node experiences higher signal attenuation, leading to a lower received RSSI. To calculate the transmission loss, the route loss model is employed, which is reflected in the received signal strength. In real-world operations, the loss model is implemented using a log-normal distribution model. This algorithm enables the estimation of signal intensity and helps determine the distance between the unnamed node and the anchor node, based on the received RSSI values.

To implement the distance-based localization technique, the first step is to analyse the network field over a 100 m area. In this scenario, the underwater sensor nodes investigate a square area that is exactly 100 m wide and 100 m deep. This analysis is conducted using MATLAB, to ensure a smooth process. The goal of this scenario is to assess how distance affects localization accuracy. The relative position of an object in space can be determined by communicating with the four anchor nodes located at the four cardinal points of the localization network. In this scenario, the network field is a 100 m square space, and there are ten mobile nodes present. The mobile node that needs to be tracked is selected as the target. The initial location of the target sensor node is randomly determined, and multiple trials are conducted to refine its location. However, only a subset of these trials is considered in this particular scenario.

**Figure 1:**
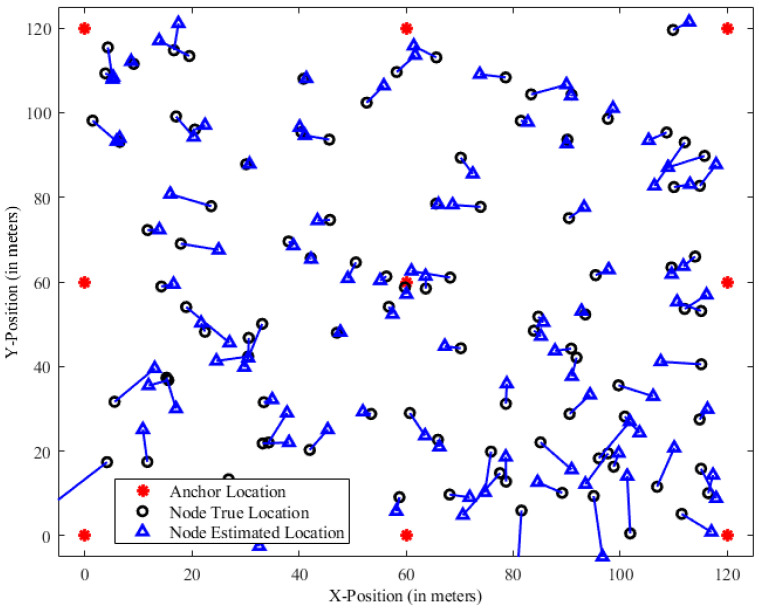
Node distribution visualization of UWSN.

To estimate the mobile node’s position, data from six trials are used initially. The beacon sensor nodes, which are connected to a reference antenna, enable the calculation of the distance between a mobile sensor node and a beacon node. This information allows for the computation of the distance between the two vertices. Figure 1 represents the visualization of the node distribution in the UWSN, illustrating the spatial arrangement of the sensor nodes and anchor nodes within the 100 m square network field. This scenario provides insights into how distance affects the accuracy of the localization technique and allows for further analysis and optimization of the system.

**Figure 2:**
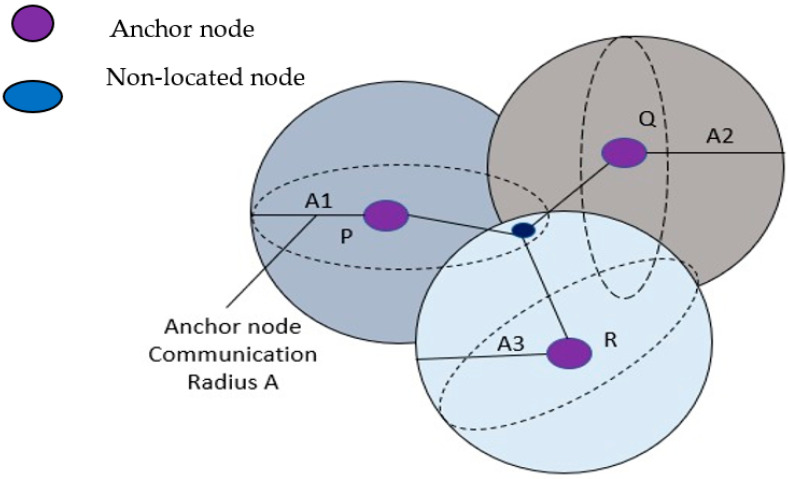
UWSN node distribution.

The shape resembles a sphere when all the nodes in a network are in constant communication with one another [31]. Signals are transmitted from connecting ties to unidentified nodes within range. The diameter of a communication path is the unit of measurement for the path’s length. Unanchored nodes receive data from the anchor node with the most reliable transmission. These nodes have a complete 360-degree view of the water depth. The sensor is represented in Figure 2. 

In the given architecture, each node incorporates a pressure detector to obtain its own independent depth data. When considering the node depth, it is possible to directly alter the *Z*-axis coordinates of the node [32]. Let us denote the coordinates of the unknown nodes as (A1, A2, and A3), and the coordinates of three anchor nodes as (A1, B1, C1), (A2, B2, C2), and (A3, B3, C3). The anchor nodes are labelled as P, Q, and R, respectively.

Now, let us assume that the coordinates of the anchor nodes are known as (Ap, Aq, and Ar). With this information, we can determine the distance between the unknown nodes and the anchor nodes [33]. The distance between two locations can be calculated using various distance measurement techniques, such as Euclidean distance or geometric distance. Let us consider the Euclidean distance formula to calculate the distance between two nodes. The Euclidean distance between two points ($x_{1}$, $y_{1},z_{1}$) and ($x_{2}$, $y_{2},z_{2}$) in 3D space is given in Equation (1):

(1) $$d=\sqrt{{\left(x_{2}-x_{1}\right)}^{2}+{\left(y_{2}-y_{1}\right)}^{2}+{\left(z_{2}-z_{1}\right)}^{2}}$$

Based on this formula, we can calculate the distance between the unknown nodes (A1, A2, and A3) and the anchor nodes (Ap, Aq, and Ar). Let us denote these distances as dPA, dQA, and dRA, respectively. Now, we have the following Equations (2)–(4) for the distance between unanchored nodes and anchor nodes:

(2) $$dPA=\sqrt{{\left(A1-Ap\right)}^{2}+{\left(A2-Aq\right)}^{2}+{\left(A3-Ar\right)}^{2}}$$

(3) $$dQA=\sqrt{{\left(B1-Bp\right)}^{2}+{\left(B2-Bq\right)}^{2}+{\left(B3-Br\right)}^{2}}$$

(4) $$dRA=\sqrt{{\left(C1-Cp\right)}^{2}+{\left(C2-Cq\right)}^{2}+{\left(C3-Cr\right)}^{2}}$$

**Figure 3:**
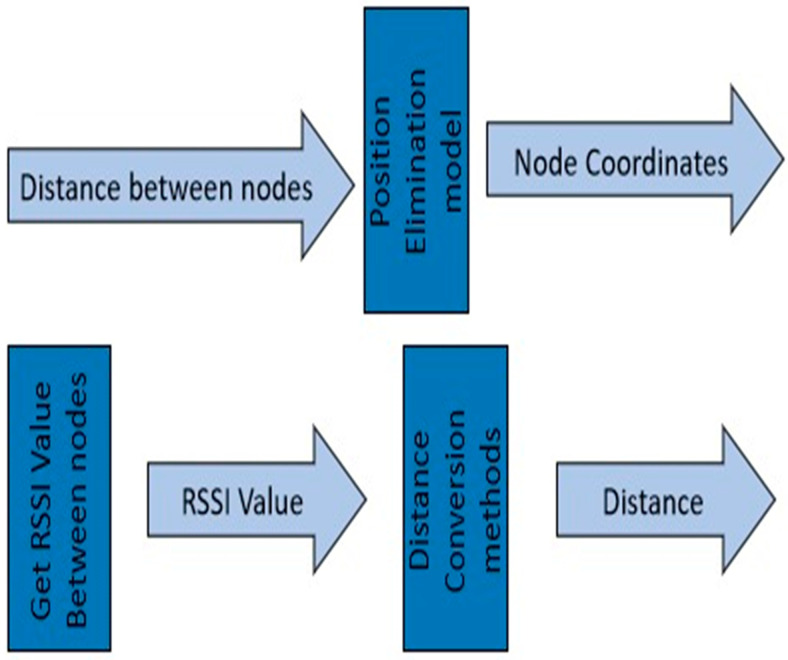
Processes involved in the RSSI.

These equations represent the distances between the unknown nodes and the anchor nodes, based on their respective coordinates [34]. By measuring these distances using the pressure detectors and solving the equations, we can estimate the coordinates (A1, A2, and A3) of the unknown nodes, and this is represented in Figure 3.

**Figure 4:**
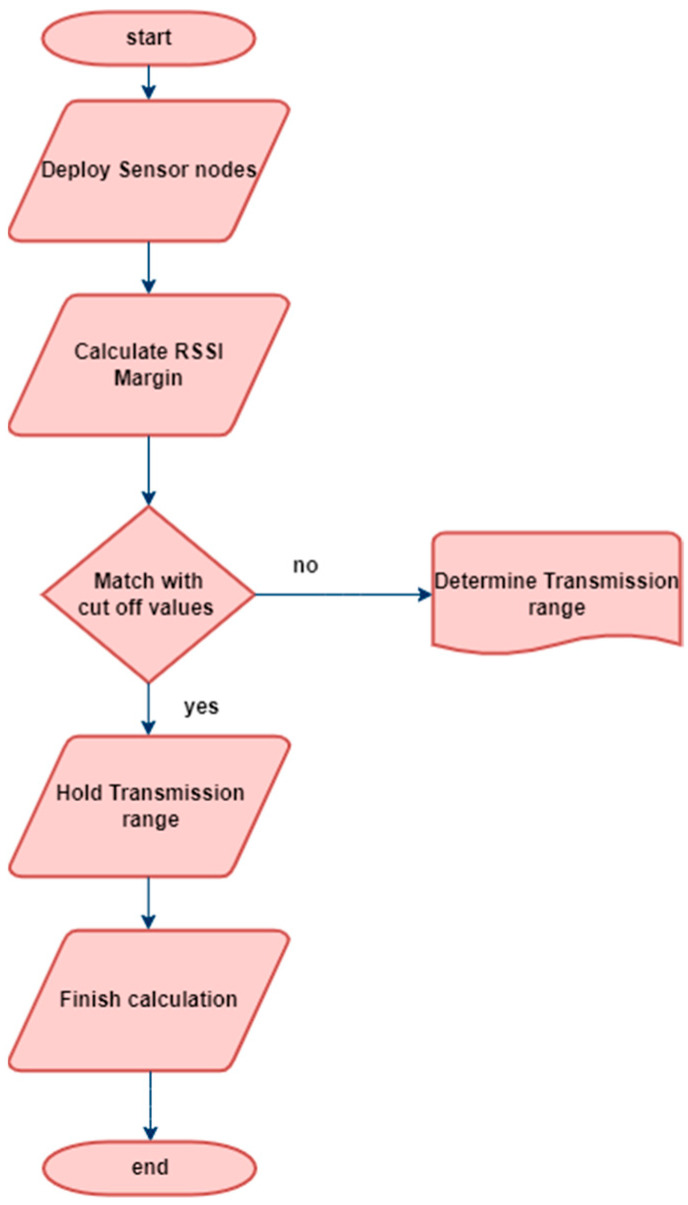
RSSI process flowchart.

The proposed algorithm for calculating the intensity of an incoming received-signal-strength-based ranging method aims to interpret the signal intensity from an anchor node at a specific distance using an unnamed node. In this scenario, the anchor node is movable and responsible for transmitting the signal. However, due to environmental factors, there is some data loss during the transmission process. As the distance increases, the unlocated node experiences higher signal attenuation, leading to a lower received RSSI. To calculate the transmission loss, the route loss model is employed, which is reflected in the received signal strength. In real-world operations, the loss model is implemented using a log-normal distribution model. This algorithm enables the estimation of signal intensity and helps determine the distance between the unnamed node and the anchor node, based on the received RSSI values; this is depicted in Figure 4.

## 4. Results and Discussion

Underwater studies have proven that the developed distance- and angle-based measures are effective, providing good support for their use in the RSSI process. The mean estimation error is used to quantify localization performance in the study “Underwater Wireless Sensor Networks with RSSI-Based Advanced Efficiency-Driven Localization and Unprecedented Accuracy”. We used RSSI-based efficient advanced localization in this study. The mean estimation error provides insight into the system’s accuracy by averaging the disparities between the estimated and ground-truth positions of sensor nodes. In terms of accuracy, the new RSSI-based localization method outperforms the baseline approaches, as evaluated by the mean estimation error. The geolocation algorithm now includes efficiency-driven methods to lower the overall impact of energy usage and communication overhead. These strategies can also have an impact on the standard error of estimates. On the mean estimation error, the effects of signal attenuation, multipath fading, and environmental factors are thoroughly studied. Limitations such as signal interference and deployment variances are recognized as opportunities for additional study and enhancements to reduce mean estimation error and increase system reliability. The achieved reduction in mean estimation error illustrates the suggested method’s practical utility under underwater conditions. Future applications of the technology in the actual world are also contemplated.

### 4.1. Analysis of Angle-Based MEEs

**Figure 5:**
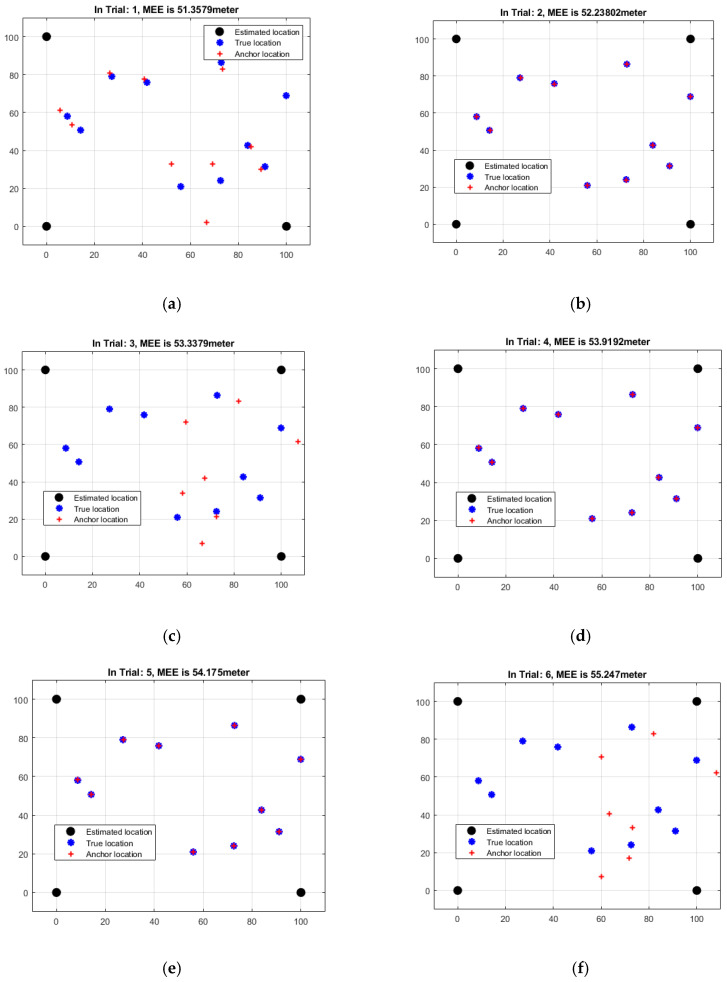
Data analysis of angle-based MEEs of six iterations: (**a**) Trial 1 (**b**) Trial 2 (**c**) Trial 3 (**d**) Trial 4 (**e**) Trial 5 (**f**) Trial 6.

The results of an examination of the mean estimation errors (MEEs) based on angles for each of the six rounds of the localization technique are shown in Table 1. The MEEs display an indicator of the average divergence between the sensor node’s estimated positions and ground-truth locations based on angle-based measurements. The MEEs varied greatly between trials, ranging from 51.3579 to 55.2470 m, according to the study which is represented in Figure 5. Different MEEs reflect varying degrees of certainty in the anticipated placements. The MEE increases from Trial 1 to Trial 6, showing that the projected locations are becoming increasingly varied. Trial 5 indicates a little drop as compared to Trial 4, indicating a transient improvement. The highest MEE was discovered in Trial 6, showing a significant difference between the observed and projected locations. These findings shed light on the shortcomings and unpredictability of angle-based approaches for locating specific positions. More research is needed to improve the accuracy and reliability of the angle-based localization technique by taking measurement errors, environmental circumstances, and the localization algorithm into account.

### 4.2. Analysis of Distance-Based MEEs

**Figure 6:**
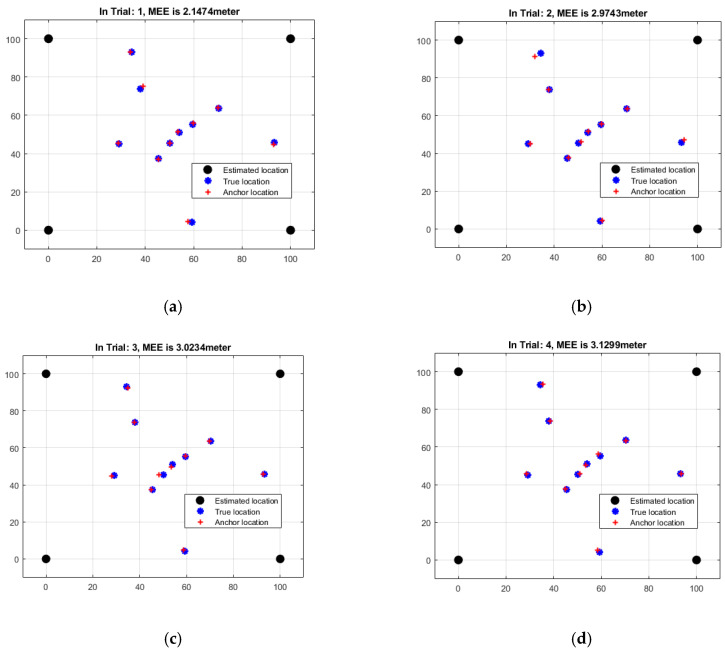

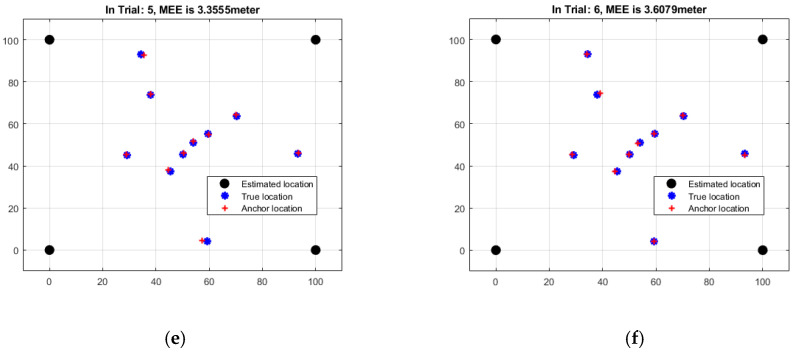
Data analysis of distance-based MEEs of six iterations: (**a**) Trial 1 (**b**) Trial 2 (**c**) Trial 3 (**d**) Trial 4 (**e**) Trial 5 (**f**) Trial 6.

Table 2 shows the results of a distance-based study of the mean estimation errors (MEEs) for each of the six rounds of the localization technique. The MEEs represent the typical difference between distance-based measurements used to anticipate sensor node locations and actual node positions. The MEEs varied between 2.1474 and 3.6079 m across the six studies, according to the study which is depicted in Figure 6. MEE variations imply that estimated positions vary in accuracy, to varying degrees. The MEE climbs from Trial 1 to Trial 6, demonstrating increasing discordance between actual and anticipated placements. This shows that when attempting to attain precise localization in this situation, distance-based assessments may have limitations. The MEEs in Table 2 are, on average, much smaller than those in Table 1 (which are based on angles). This would imply that distance-based measurements provide substantially higher precision, albeit with considerable opportunity for error. To improve the accuracy and reliability of the distance-based localization strategy, more research and analysis of a variety of parameters, such as measurement errors, environmental factors, and the localization algorithm, is necessary.

### 4.3. Analysis of RSSI-Based MEEs

**Figure 7:**
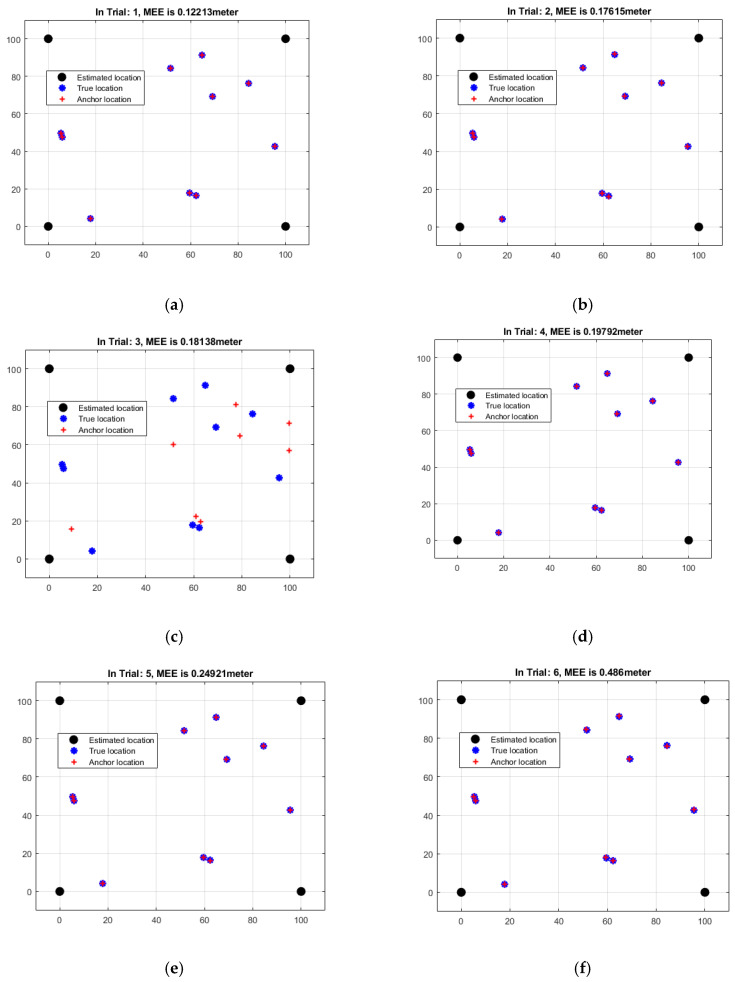
Data analysis of RSSI-based MEEs of six iterations: (**a**) Trial 1 (**b**) Trial 2 (**c**) Trial 3 (**d**) Trial 4 (**e**) Trial 5 (**f**) Trial 6.

Table 3 shows a breakdown of the mean estimation errors (MEEs) based on the RSSI distance for each of the six rounds of localization. The MEEs represent the average difference between the predicted sensor node positions and the ground-truth positions as established by RSSI’s distance-based measurements. The study discovered that MEEs vary substantially from experiment to experiment, ranging from 0.12213 to 0.48601 m which is represented in Figure 7. MEE variations imply that estimated positions vary in accuracy, to varying degrees. The MEE grows rapidly from Trial 1 to Trial 6, suggesting that the projected positions deteriorate. Table 3’s MEEs are far closer to reality than those of Table 1 and Table 2, which are based on angles and distances. This suggests that the RSSI distance-based metrics provide more precision and accuracy in the overall localization process. However, even with lower MEEs, there is still significant discordance between the projected and actual positions. This is a critical factor to consider. To increase the accuracy and reliability of the RSSI distance-based localization strategy, more research, optimization, and thorough examination of aspects such as measurement errors, ambient circumstances, and the localization algorithm are required.

### 4.4. Comparative Analysis

The distance between sensor and anchor nodes must be measured for network localization. As a result of using this strategy, the network takes on a square shape, with nodes separated by 100 m along the perimeter. Because there are no fixed sensor nodes present, mobile sensor nodes are free to traverse the allotted space at their leisure. The network has fourteen nodes, four of which are fixed and ten of which are mobile. Each sensor node in the network has the ability to communicate with one of the anchor nodes. These anchor nodes are deliberately located at each of the network’s four corners. These devices are 90% accurate in terms of distance estimation, with an error ratio of 0.1 m. This concept is best illustrated by an accuracy of one metre or near it. Before proceeding with real distance measurements between sensor nodes, it is important to make a calculation to prove that the sensor nodes are not evenly distributed. After installing the sensor nodes, the procedure of collecting MEEs and assessing the findings is repeated several times. This procedure has been extensively tried and tested; data from six separate experiments on angle, distance, and RSSI are considered. The MEEs oscillate between 51.3579 m and 55.2470 m, with respect to angular measurements, MEEs’ distance-based measurements ranged from 2.1474 to 3.6079 m, and MEEs range from 0.12213 to 0.4860 m for RSSI-based measurements, as depicted in Table 4.

Table 4 shows the mean estimation errors (MEEs) for each of the three measurement methods—angle of arrival (AOA), time difference of arrival (TDOA), and received signal strength indication (RSSI). The MEEs illustrate how far the sensor nodes’ estimated locations differ from where they actually are on the map. The MEEs’ AOA could range from 51.3579 to 55.2470 m. MEE variations can be understood as variances in the dependability of predicted positions generated from angle-based measurements. The MEEs’ TDOA ranges from 2.1474 to 3.6079 m. These MEEs show the spread of distance-based position estimates based on time-of-arrival data. The RSSI values of the MEEs range from 0.12213 to 0.48601 m. These MEEs depict the errors in estimated positions based on received signal strength indicators (RSSI). When MEEs estimated using RSSI measurements are compared to MEEs calculated using AOA and TDOA, it is evident that the former are substantially smaller. This implies that RSSI-based measurements provide more precision and accuracy during localization.

## 5. Conclusions and Future Scope

This article explores and covers the distance-dependent RSSI localization approaches. After determining the positions of the subsea nodes, the MEEs may be approximated. To take distance measurements, a network field of one hundred metres on each side and one hundred metres in total length was required. Mobile sensor nodes were given the freedom to wander freely throughout the networked space and engage in two-way communication with one another. The four surviving nodes in the network, known as the anchor nodes, were the only nodes in the network that did not migrate from their initial position at any point in time. To gather readings from the MEE, the location of a sensor node was chosen at random, and the process occurred while the readings were being obtained. A number of experiments were performed following the arbitrary placement of the sensor nodes; however, only a subset of these trials were considered for the sake of the baseline scenario. This is because the experiment began with the arbitrary placement of the sensor nodes. We carried out research and analysis on a total of six different trial count permutations in order to compute the MEEs. In terms of angle measurements, the MEEs have a range of 51.3579 to 55.2470 m. The distance-based measurements of the MEEs range from 2.1474 to 3.6079 m, whereas the RSSI-based observations range from 0.12213 to 0.4860 m. When MEEs calculated with AOA and TDOA are compared to MEEs estimated with RSSI measurements, it is obvious that the latter are substantially bigger. This implies that RSSI measurements provide a higher level of precision and accuracy during the localization process. The future scope involves advancing the understanding and capabilities of distance-dependent RSSI localization, addressing limitations, and exploring new avenues to achieve higher precision and accuracy in underwater localization scenarios.

## Figures and Tables

**Table 1:** Data analysis of angle-based MEEs for six iterations.

Trial	Distance (m)
1	51.3579
2	52.2380
3	53.3379
4	53.9192
5	54.1750
6	55.2470

**Table 2:** Data analysis of distance-based MEEs for six iterations.

Trial	Distance (m)
1	2.1474
2	2.9743
3	3.0234
4	3.1299
5	3.3555
6	3.6079

**Table 3:** Data analysis of RSSI distance-based MEEs for six iterations.

Trial	Distance (m)
1	0.12213
2	0.17615
3	0.18138
4	0.19792
5	0.24921
6	0.48601

**Table 4:** Data analysis of MEEs for six iterations.

Trial	Distance (m)		
	AOA	TDOA	RSSI
1	51.3579	2.1474	0.12213
2	52.2380	2.9743	0.17615
3	53.3379	3.0234	0.18138
4	53.9192	3.1299	0.19792
5	54.1750	3.3555	0.24921
6	55.2470	3.6079	0.48601

## Data Availability

The datasets used during the current study are available from the corresponding author upon reasonable request.

## Abbreviations

Underwater wireless sensor networks (UWSNs)
TDOA majorization-minimization (T-MM)
Majorization-minimization (MM)
Squared position error bound (SPEB)
Global positioning system (GPS)
Infrared (IR)
Changeable transmission power-based sparsity-conscious energy-efficient clustering (CTP-SEEC)
Angle of arrival (AOA)
Time difference of arrival (TDoA)
Received signal strength indicator (RSSI)
Mean estimation error (MEE)
Equivalent Fisher information matrix (EFIM)
Line-of-sight (LOS)
Wireless sensor network (WSN)
Autonomous underwater vehicles (AUVs)
Transmission power level (TPL)
